# Disruption of Pathological Patterns in a Young Population with Dysfunctional Breathing

**DOI:** 10.1155/2020/9614574

**Published:** 2020-09-19

**Authors:** Ji-Myung Ok, Young-Jae Park

**Affiliations:** ^1^Department of Human Informatics of Korean Medicine, Graduate School, Kyung Hee University, Seoul, Republic of Korea; ^2^Department of Biofunctional Medicine and Diagnostics, College of Korean Medicine, Kyung Hee University, Seoul, Republic of Korea; ^3^Department of Diagnosis and Biofunctional Medicine, Kyung Hee University Hospital at Gangdong, Seoul, Republic of Korea

## Abstract

Dysfunctional breathing (DB) is characterized by abnormal breathing patterns and often results from psychogenic causes in the absence of organic diseases. Although acupuncture and herbal treatments have been suggested as alternative therapies for DB, few studies have addressed the relationship between DB and pathological patterns from a diagnostic perspective. We asked 237 college students (130 men aged 21.4 ± 1.9 years; 107 women aged 21.4 ± 3.0 years) to complete the Korean version of the General Health Questionnaire-30 (K-GHQ-30) and four validated pathological pattern questionnaires regarding qi and yin deficiencies, phlegm, and cold-heat patterns. The Korean version of the Nijmegen Questionnaire was used to classify participants into DB and non-DB groups. Effects of age, gender, and DB on pathological patterns were examined using simple regression and two-way MANCOVA models. Age had an effect on all pattern scores except heat pattern scores (*β*: 0.154–0.098). DB group showed a moderate main effect (*η*^2^ = 0.167) on pathological patterns, while gender showed a minimal main effect (*η*^2^ = 0.096); qi and yin deficiencies, phlegm, and cold-heat pattern scores in DB and female groups were higher than those in non-DB and male groups. The K-GHQ-30 scores showed significant positive correlations with the pathological pattern scores (*r*: 0.243–0.533), indicating that disruption of pathological patterns could be associated with patients' psychological disturbance. In conclusion, these questionnaires may help to identify pathological patterns related to DB and determine individually tailored alternative therapies.

## 1. Introduction

Dysfunctional breathing (DB) is characterized by abnormal breathing patterns that result either from psychogenic causes in the absence of organic disease (primary DB) or from cardiopulmonary or neurological diseases such as asthma and heart failure (secondary DB) [1]. Frequently, DB is associated with hyperventilation syndrome (HVS) in which the concentration of arterial carbon dioxide (CO_2_) is excessively lowered beyond normal physiological levels, resulting in a wide range of respiratory, neurological, and psychological problems [2]. In the absence of underlying disease, HVS is classified as idiopathic hyperventilation and may present as chronic asymptomatic hyperventilation and respiratory alkalosis [3].

To identify individuals with DB, the Nijmegen Questionnaire (NQ), the Hyperventilation Provocation Test, and cardiopulmonary exercise tests have been utilized [4]. Among these, the NQ is the most widely used instrument because of its convenience and cost-effectiveness. The NQ consists of 16 items to assess symptoms such as palpitation, chest pain, dyspnea, light-headedness, anxiety, and paresthesia [4]. The original NQ has been translated into many languages including Greek, Norwegian, Persian, and Korean [5–8].

The prevalence of DB is reportedly 5–11% in the general population [9, 10] and as high as 83% in subjects with anxiety [11]. DB is often overlooked and untreated, resulting in unnecessary suffering [12, 13]. Moreover, the symptoms of DB may be incorrectly attributed to asthma [14]. Some studies have reported that DB reduces quality of life [8, 15, 16].

Breathing retraining (BR), in which patients are encouraged to reduce their respiratory rate and use diaphragmatic breathing, is generally accepted as the primary option for DB treatment [1, 17]. BR along with relaxation therapy based on yoga techniques has been reported to be more effective than relaxation therapy alone [18]. However, the best therapeutic strategy for patients with DB, especially for those with primary DB, has not yet been established because DB is a complex condition involving respiratory, biochemical, biomechanical, psychological, and social aspects [1]. Therefore, there has been high demand for alternative therapies for DB such as acupuncture or herbal treatment, which are safe and cost-effective, raise little risk of dependence, and have a psychosomatic effect. Gibson et al. reported that acupuncture was effective in alleviating the anxiety associated with DB [19]. Other studies have reported that herbal prescriptions have been effective for DB treatment [20–22]. However, a limitation of these studies is that each of them related DB to only one pathological pattern. For example, Cai and Wang related DB to qi deficiency and prescribed Shenzhezhenqi Pill for DB patients [20], while Li et al. and Hu and Zuo related DB to qi stagnation and suggested Yueju Pill and Shexiang Baoxin Pill, respectively [21, 22]. In terms of acupuncture, Gibson et al. did not consider the pathological pattern and used the Hospital Anxiety and Depression Scale to evaluate the efficacy of acupuncture treatment on HVS-related anxiety [23]. Given these discrepancies in the literature, it is important to examine which pathological patterns [19], DB may be related to before prescribing alternative therapy. In this study, we hypothesized that DB may affect the clinical severity of multiple pathological patterns because of its multifaceted nature, and the aim of our study was to elucidate the relationships between DB and pathological patterns. This work may help practitioners tailor individualized therapy with acupuncture or herbal prescriptions for DB patients and maximize the clinical efficacy of alternative treatments combined with BR.

## 2. Subjects and Methods

### 2.1. Participants

A total of 130 men (21.35 ± 1.87 years old) and 107 women (21.41 ± 3.02 years old) participated in this study; all were college students at Kyung Hee University. The subjects were asked to complete the Korean version of the NQ (KNQ) [8]; four pathological pattern questionnaires: the Lao Juan Questionnaire (LJQ) [24], Yin Deficiency Scale (YDS) [25], Phlegm Pattern Questionnaire (PPQ) [26], and Cold-Heat Pattern Questionnaire (CHPQ) [27]; and the Korean version of the General Health Questionnaire-30 (K-GHQ-30) [28]. Based on self-reports, young college students without any physical or mental problems that can prevent them from having a normal college life were included in this study, while the students who self-reported any respiratory or psychiatric problems relating to DB were excluded. This study was approved by the institutional review board of University-Industry Cooperation at Kyung Hee University (KHSIRB-15-010). All subjects provided informed consent.

### 2.2. Measurements: KNQ, LJQ, PPQ, YDS, CHPQ, and K-GHQ-30

The NQ consists of 16 items that are rated on a five-point Likert scale (0 never, 1 rarely, 2 sometimes, 3 frequently, and 4 very frequently). The total NQ score ranges from 0 to 64 points; a higher score indicates more severe symptomatic hyperventilation. The KNQ has been previously validated, and its cross-cultural translation into Korean has also been validated [8].

Pathological pattern questionnaires were chosen based on a review of previous studies. Lao Juan, the Chinese term for overexertion, is one of the main factors resulting in qi deficiency. The LJQ includes 19 items assessing symptoms such as tiredness, weakness of the limbs, frequent common colds, weak voice, spontaneous sweating, fever, shortness of breath, indigestion, and poor appetite [24]. This questionnaire was included in our study because some of these symptoms are observed in DB, such as weak voice and shortness of breath, and the Shenzhezhenqi Pill, a treatment for qi deficiency, has been reported to be effective in treating DB [20]. The YDS estimates the clinical severity of yin deficiency; it consists of 27 items assessing symptoms such as dry mouth, facial flush in the afternoon, night sweats, tinnitus, hot soles at night, bone steaming, lower back pain, dull pain in the ankles or knees, frequent urination, and hard stools [25]. As certain DB-related symptoms, such as shortness of breath, tingling in the fingers, tight feelings around the mouth, and dizzy spells, are related either to yin deficiency or to hyperactivity of liver yang due to yin deficiency, the YDS was included in this study [29]. The PPQ, which measures the clinical severity of the phlegm pattern, consists of 25 items that assess symptoms including palpitations, feeling of pressure in the chest, dizziness, sputum, feeling as if there is a foreign body in the throat, cough, joint pain, shortness of breath, flank pain, indigestion, feeling of abdominal fullness, sickness, and rumbling sounds in the abdomen [26]. As certain DB-related symptoms such as palpitations, anxiety, dizzy spells, and a feeling of abdominal fullness are related to the phlegm pattern, the PPQ was included in this study. The LJQ, PPQ, and YDS have previously been validated and are rated on a 7-point Likert scale (1 strongly disagree and 7 strongly agree).

The CHPQ estimates the clinical severity of cold and heat patterns; it consists of 10 cold pattern-related and 10 heat pattern-related items [27]. The cold pattern-related items include aversion to cold, preference for heat, abdominal coldness, coldness of the limbs, cold pain, and pale face. Heat pattern-related items include preference for cold, body heat, feverish pain, thirst, thick yellow sputum, and hot breath. The CHPQ has been previously validated [27]. In this study, we hypothesized that an imbalance between cold and heat patterns may be apparent among young people with DB. The CHPQ is rated on a 5-point Likert scale (1 strongly disagree and 5 strongly agree). A higher score represents greater severity of the corresponding pathological pattern.

The General Health Questionnaire (GHQ) consists of 30 items to evaluate health-related quality of life level, especially that related to psychological disorders [28]. The K-GHQ-30 has previously been validated and is rated on a 4-point Likert scale (1 not at all and 4 much more than usual) [28]. A higher score represents severe aggravation of one's quality of life associated with a psychological disorder. The correlations between the K-GHQ-30, KNQ, LJQ, PPQ, YDS, and CHPQ scores were calculated to examine how the disruption of pathological patterns in the DB state was related to one's psychological disorder.

### 2.3. Statistical Analysis

Subjects who scored 23 points or more on the KNQ were assigned to the DB group, whereas those who scored fewer than 23 points were assigned to the non-DB group, according to the protocol described in a previous study [4]. The effect of age on LJQ, YDS, PPQ, and CHPQ scores was examined using simple regression modeling. Pearson's correlations between the four questionnaire scores were examined. Since there was a significant effect of age, significant correlations were identified between the questionnaire scores, and there was equal covariance across the gender and DB groups, two-way MANCOVA with age as a covariate was performed. In the MANCOVA test, gender (male and female) and group (DB group and non-DB group) were defined as within-subject factors, and the four questionnaire scores were defined as between-subject factors. The main effects of between-subject factors were examined using Wilks' Lambda, and effect sizes were calculated using partial eta squared (*η*^2^): no effect (*η*^2^ < 0.04), minimal effect (0.04 < *η*^2^ < 0.25), moderate effect (0.25 < *η*^2^ < 0.4), and strong effect (*η*^2^ > 0.64) [30]. All statistical analyses were performed using SPSS 21 (SPSS Inc., Chicago, IL, USA). *p* values <0.05 were considered significant.

## 3. Results

Descriptive data regarding the LJQ, YDS, PPQ, CHPQ, and KNQ scores are presented in Table 1.  Table 2 shows the effects of age on the five pathological pattern scores. Except for the heat score of the CHPQ, an effect of age was identified on all pattern questionnaire scores, so age was considered as a covariate for the two-way MANCOVA model described below.  Table 3 shows the Pearson's correlations between the K-GHQ-30 and five pattern questionnaire scores. All the scores showed significant positive correlation coefficients, ranging from 0.191 to 0.824. Moreover, Box's test of equality of covariance revealed equal covariance of the dependent variables across gender and DB groups (Box's *M* = 62.175, *p* = 0.085). Therefore, a two-way MANCOVA model, in which gender and DB groups were set as within-subject factors, was used to examine the main effects of DB and gender on pathological pattern scores. This revealed that gender (*p* < 0.001, *η*^2^ = 0.096; minimal effect) and DB group (*p* < 0.001, *η*^2^ = 0.167; moderate effect) had significant main effects on the pathological pattern scores. However, there was no significant interaction between gender and DB groups (*p* < 0.736, *η*^2^ = 0.012; no effect).

Table 4 shows the differences in the five pathological pattern scores between the DB and non-DB groups, and between genders. Among the pattern scores, the LJQ, PPQ, and cold score of the CHPQ showed gender effects, while gender effects were not observed for the YDS and the heat score of the CHPQ. All of the pattern scores showed DB group effects, indicating that pattern scores in the DB group were higher than those in the non-DB group.

## 4. Discussion

In this study, we used MANCOVA to examine the effect of DB on pathological patterns; this analysis was selected because an effect of age was observed on all pathological pattern scores except for the heat score of the CHPQ. Moreover, pathological pattern scores showed significant correlations with each other. The effect of age found in this study is consistent with a previous study that found an effect of age on the YDS and PPQ scores of young subjects [31]. Interestingly, the *β* values of the LJQ, YDS, and PPQ scores and the cold score of the CHPQ were all similar to each other (*β*: 0.154–0.198). Therefore, the effect of age on pathological patterns should be considered when examining the relationships between DB and pathological patterns. However, it should be emphasized that this study assessed a relatively young population. Therefore, it is necessary to examine whether the effect of age on pathological patterns is also seen in older groups. In terms of the relationships between pathological patterns, scores related to a “deficiency” pattern, such as LJQ and YDS scores, showed positive correlations with scores from questionnaires such as the PPQ that are related to an “excess” pattern. This indicates that, in a young population, pathological patterns may not be formed in isolation, but there may instead be a complex picture in which deficiency patterns may be combined with other deficiency or excess patterns.

The main finding of our study is that all pathological pattern scores were higher in the DB group than in the non-DB group. Furthermore, qi deficiency, phlegm, and cold pattern scores were higher in females than in males, indicating an effect of gender. There was no interaction found between DB group and gender. Therefore, our study suggests that the clinical severity of pathological patterns may be higher in the DB group than in the non-DB group, despite the susceptibility of females to conditions in which pathological patterns are aggravated. As mentioned above, previous studies on DB have been limited to considering a single pathological pattern, such as qi deficiency or qi stagnation [20–22]. According to our hypothesis that DB may be related to multiple pathological patterns, our study results showed that the aggravation in pathological patterns in the DB group was not limited to a single pattern, but rather included all of the four pathological patterns studied. This result reflects the general reduction in quality of life (QoL) subscale scores seen among DB patients [8]. Considering the positive correlations between the K-GHQ-30 and the four pathological pattern scores, it appears that disruption of pathological patterns in a young population with DB may be associated with psychological disturbance. In addition to the deficiency and phlegm pattern scores, cold and heat scores were both higher in the DB group than in the non-DB group. This indicates that cold and heat patterns are not mutually exclusive: there can be a complex pattern of cold-heat in which the heat pattern is located in the upper body or in the exterior, while the cold pattern is simultaneously located in the lower body or in the interior [28]. Altogether, complex pathological patterns constituting qi and yin deficiency, phlegm, and cold and heat patterns may develop in DB. However, it is important to emphasize a limitation of this cross-sectional study: it was not possible to tell whether disturbance in one pathological pattern may have resulted in aggravation of other patterns or whether the pathological patterns were simultaneously aggravated along with DB.

Gender had a minimal effect size (*η*^2^ = 0.096), while DB group had a moderate effect size (*η*^2^ = 0.167). Therefore, the differences in pathological pattern severity in a young population may be affected more by DB group than by gender. In order to maximize the efficacy of alternative medicine treatment in patients with DB, the utilization of validated pattern questionnaires may aid the identification of complex pathological patterns related to DB and thus help practitioners to develop individually tailored prescriptions of herbs or acupuncture.

This study has certain limitations. It only included young people in Seoul, Korea; the results may therefore not be generalizable to all populations. Moreover, our study did not include data on arterial CO_2_ levels, and it is unclear whether there may have been differences in the association with pathological patterns between symptom-based and arterial CO_2_ level-based DB data. Further studies will be necessary to address these limitations. Furthermore, a longitudinal study is required to examine whether combined therapy with acupuncture or herbs along with BR may be more effective than BR therapy alone.

## 5. Conclusion

In this study, we examined the effect of DB on pathological patterns in a young population. Our study found an effect of age on qi and yin deficiencies, phlegm, and cold pattern scores. MANCOVA showed that qi and yin deficiencies, phlegm, and cold-heat pattern scores in the DB and female groups were higher than those in the non-DB and male groups. In conclusion, the utilization of validated pattern questionnaires may aid the identification of pathological patterns related to DB and the development of individually tailored alternative therapies for patients with DB.

## Figures and Tables

**Table 1:** Scores on the KNQ, the K-GHQ-30, and the pattern questionnaires.

Scale	Score (mean ± SD)	
	Men (*n* = 130)	Women (*n* = 107)
KNQ	13.93 ± 9.43	17.65 ± 9.02
K-GHQ-30	31.47 ± 11.58	34.96 ± 12.56
LJQ	60.27 ± 19.01	67.97 ± 20.04
YDS	71.13 ± 23.20	74.20 ± 25.88
PPQ	71.56 ± 22.78	82.48 ± 25.36
CS of the CHPQ	29.31 ± 8.68	33.32 ± 8.96
HS of the CHPQ	31.74 ± 9.14	30.11 ± 9.92

KNQ: Korean version of the Nijmegen Questionnaire, K-GHQ-30: the Korean version of the General Health Questionnaire, LJQ: Lao Juan Questionnaire, YDS: Yin Deficiency Scale, PPQ: Phlegm Pattern Questionnaire, CS: cold syndrome, HS: heat syndrome, CHPQ: Cold-Heat Pattern Questionnaire, SD: standard deviation.

**Table 2:** Effects of age on LJQ, PPQ, YDS, and CHPQ scores.

Questionnaire (score)	Adj. *R*^2^	*β*	SE	*t*	*p* value
LJQ	0.035	0.198	0.518	3.089	0.002
YDS	0.024	0.167	0.642	2.600	0.010
PPQ	0.021	0.159	0.646	2.473	0.014
Cold score of the CHPQ	0.020	0.154	0.237	2.388	0.018
Heat score of the CHPQ	0.004	0.093	0.252	1.428	0.155

LJQ: Lao Juan Questionnaire, YDS: Yin Deficiency Scale, PPQ: Phlegm Pattern Questionnaire, CHPQ: Cold-Heat Pattern Questionnaire, SE: standard error.

**Table 3:** Pearson's correlations between K-GHQ-30, LJQ, YDS, PPQ, and CHPQ scores.

Scale (score)	K-GHQ-30	LJQ	YDS	PPQ	Cold score of the CHPQ	Heat score of the CHPQ
K-GHQ-30	1					
LJQ	0.533^*∗∗*^	1				
YDS	0.469^*∗∗*^	0.760^*∗∗*^	1			
PPQ	0.496^*∗∗*^	0.824^*∗∗*^	0.822^*∗∗*^	1		
Cold score of the CHPQ	0.293^*∗∗*^	0.533^*∗∗*^	0.458^*∗∗*^	0.507^*∗∗*^	1	
Heat score of the CHPQ	0.243^*∗∗*^	0.490^*∗∗*^	0.555^*∗∗*^	0.475^*∗∗*^	0.191^*∗∗*^	1

K-GHQ-30: the Korean version of the General Health Questionnaire, LJQ: Lao Juan Questionnaire, YDS: Yin Deficiency Scale, PPQ: Phlegm Pattern Questionnaire, CHPQ: Cold-Heat Pattern Questionnaire. ^*∗∗*^*p* < 0.01.

**Table 4:** Differences in the four pattern questionnaire scores between the DB and non-DB groups, and between genders (participants, *n* = 237).

Scale	Subscale	Group	Gender		Factor	*F*	*p* value
			Male	Female			
LJQ		DB	70.67 ± 21.51	81.47 ± 18.07	Gender	7.544	0.006
					DB/non-DB	30.179	<0.001
		Non-DB	57.92 ± 17.67	62.71 ± 18.31	Gender *∗* (DB/non-DB)	1.124	0.290

YDS		DB	86.92 ± 23.10	90.47 ± 22.66	Gender	0.273	0.602
					DB/non-DB	34.253	<0.001
		Non-DB	67.56 ± 21.78	67.86 ± 24.35	Gender *∗* (DB/non-DB)	0.206	0.650

PPQ		DB	88.71 ± 20.73	100.63 ± 23.69	Gender	8.245	0.004
					DB/non-DB	45.215	<0.001
		Non-DB	67.68 ± 21.47	75.40 ± 22.40	Gender *∗* (DB/non-DB)	0.376	0.540

CHPQ	Cold	DB	33.21 ± 8.53	36.87 ± 6.86	Gender	7.141	0.008
					DB/non-DB	12.729	<0.001
		Non-DB	28.43 ± 8.51	31.94 ± 9.33	Gender *∗* (DB/non-DB)	0.003	0.960
	Heat	DB	34.21 ± 9.44	35.13 ± 10.28	Gender	0.547	0.460
					DB/non-DB	11.695	0.001
		Non-DB	31.18 ± 9.02	28.16 ± 9.11	Gender *∗* (DB/non-DB)	1.865	0.173

^∗^An interaction between gender and the DB group. LJQ: Lao Juan Questionnaire, YDS: Yin Deficiency Scale, PPQ: Phlegm Pattern Questionnaire, CHPQ: Cold-Heat Pattern Questionnaire, DB: dysfunctional breathing.

## Data Availability

The Excel-fomat data used to support the findings of this study are included within the supplementary materials.

## Supplementary Materials

Supplementary Materials The Excel-format dataset used in this study.
